# Low Colonisation Rate of Candida auris in Patients of Intensive Care Units: A Study From Central India

**DOI:** 10.7759/cureus.86823

**Published:** 2025-06-26

**Authors:** Surabhi Sahay, Archana Keche, Padma Das, Ujjawala Gaikwad, Kumar Vikram

**Affiliations:** 1 Ophthalmology, All India Institute of Medical Sciences, New Delhi, New Delhi, IND; 2 Microbiology, All India Institute of Medical Sciences, Raipur, Raipur, IND; 3 Microbiology, ESI-PGIMSR, ESIC Medical College and Hospital, Kolkata, IND

**Keywords:** candida auris, candida species, colonisation, icu, screening

## Abstract

Introduction

Invasive candidiasis is associated with increased morbidity and mortality in critically ill patients. *Candida auris (C. auris)* is a growing concern in healthcare settings, especially in intensive care units (ICUs), due to its rapid spread, limited treatment options, and high mortality risk, posing a substantial challenge for physicians. In this study, we aimed to estimate the prevalence of *C. auris *colonization in patients admitted to ICUs of a tertiary care centre in central India.

Methods

A composite swab from the axilla and groin of patients at the time of admission and one week after admission was collected and subjected to culture and identification by conventional methods and using the Vitek 2 Yeast Identification System. Data from the case record forms were compiled in an Excel sheet (Microsoft® Corp., Redmond, WA) and analysed. Descriptive statistics, including frequency, mean, and standard deviation, were calculated for the demographic data. All statistical analyses were performed using Epi Info (version 7.2.5.0; Centers for Disease Control and Prevention, Atlanta, GA).

Results

Out of the 67 patients enrolled in the study, follow-up samples after one week of admission were only possible for 37 of the patients. Overall, a total of 18 *Candida* isolates were obtained from 67 patients, out of which, only one isolate (5.55%) was identified as *C. auris*. Other species of *Candida* were *Candida glabrata* (13), *Candida parapsilosis* (three), and *Candida tropicalis* (one). *Candida* colonisation was seen more in patients on meropenem and amoxicillin-clavulanate acid (p=0.02).

Conclusion

The low detection rate of *C. auris* colonisation among ICU patients in this study suggests limited spread within the facility during the study period. However, the overall *Candida *colonisation, particularly among patients receiving broad-spectrum antibiotics, highlights the importance of antimicrobial stewardship and active surveillance.

## Introduction

*Candida auris* infections pose a significant threat in intensive care units (ICUs), given their potential for outbreaks, resistance to multiple drugs, and high mortality rates, complicating patient care [1]. It was first identified in 2009 in Japan after its isolation from the external ear canal of a patient by analyses of the 26S rDNA D1/D2 domain, nuclear ribosomal DNA ITS region sequences, and chemotaxonomic studies [2]. *C. auris* frequently colonises the skin specifically axilla and groin in addition to other sites, such as the nares, oropharynx, external ear canal, vagina, and rectum in hospitalised patients [3]. Moreover, colonising specimens are commonly associated with the aggregative phenotype of *C. auris* and have demonstrated significant biofilm production abilities [4]. Because of its biofilm-forming abilities, it can adhere to polymeric substances (commonly present in ICUs) and exhibit resistance to antifungal agents [5]. *C. auris* can cause serious bloodstream infections affecting the blood, heart, or brain and can even cause death [6]. Currently, it has gained importance because it is resistant to commonly used antifungal agents, such as azoles, polyenes, and even echinocandins, and the mortality rate is 33-72% in candidemia patients [6]. Prolonged ICU stays, prior exposure to broad-spectrum antibiotics, and invasive medical procedures place both pediatric and adult patients at a high risk of developing *C. auris* infection [7]. Hospital surveillance studies have indicated that this opportunistic pathogen can be acquired from both dry and moist surfaces within healthcare environments. The prolonged survival of *C. auris *on hospital surfaces is attributed to its production of virulence factors, such as hydrolytic enzymes, along with its capacity to form biofilms, which facilitate persistent colonisation on human skin and in the surrounding environment [8]. Therefore, the development and use of more effective disinfectants and antiseptics are essential to strengthen hospital infection control practices [9].

The extent of the problem, as suggested by previous studies, makes it vital to diagnose *C. auris* infections and colonisations early and accurately among patients admitted to critical care units. Most patients admitted to ICUs are critically ill and have a weak immune response due to poor health conditions. Hence, it is logical to assume that colonisation can lead to disseminated infections, which could be dangerous for these patients. This study detected the rate of *C. auris *colonisation in admitted patients in ICUs. The presence of *C. auris* colonisation in patients will alert us to the need for cautious use of transmission-based precautions. These findings may also guide us regarding the need for antifungal prophylaxis. This study will also guide us to recommend a protocol for routine screening of *C. auris* in critical care areas of hospitals and to develop policies regarding the treatment of colonisation, which poses a risk of transmission and, thus, the subsequent prevention and management of infection.

## Materials and methods

Study design and population

This is a cross-sectional observational study conducted by the Department of Microbiology as part of a short-term studentship (STS) project (project no 2020-04198) under the Indian Council of Medical Research (ICMR) for a period of four months from March 2021 to June 2021. All new patients, irrespective of age or gender, admitted to the General Medicine Intensive Care Unit were included in the study. Patients with prior antifungal therapy were excluded from the study. Appropriate written informed consent was taken from the patients or their relatives. The study was approved by the Institute's Ethics Committee (letter no. 1185/IEC-AIIMSRPR/2020 dated 08.08.2020).

Sample collection

With all aseptic precautions, a composite swab of the patient’s bilateral axilla and groin was collected twice, first at the time of admission and second, after one week of admission, and it was submitted to the microbiology laboratory. Sample collection was done in accordance with the procedure described by the CDC [3].

Laboratory procedures

The swabs were inoculated on two culture media sets, each set containing one plain Sabouraud dextrose agar (SDA) and one SDA with chloramphenicol. One set was incubated at 37°C and another at 25°C in a biochemical oxygen demand (BOD) incubator. The culture plates were examined after 48 hours for fungal growth. Identification of *Candida *species was done by standard mycology laboratory procedures, which included Gram stain, Germ tube test, Dalmau plate culture, growth in the presence of 0.01% or 0.1% cycloheximide, sugar assimilation, and sugar fermentation method, and by inoculating it on CHROMagar [10]. Identification of *C. auris* was done by inoculation on yeast extract peptone dextrose agar with 12.5% NaCl and incubating at 42°C for five days [11]. Identification was also done with Vitek 2 Yeast Identification System (version 8.01; bioMérieux, Marcy l'Etoile, France) by using the YST Card to support the organism identification. Matrix-assisted laser desorption/ionisation time-of-flight (MALDI-TOF) could not be done due to its unavailability.

Data collection and statistical analysis

Patient demographics and clinical data were documented as per the case record form. Data from the case record forms were compiled in an Excel sheet (Microsoft® Corp., Redmond, WA) and analysed. Descriptive statistics, including frequency, mean, and SD, were calculated for the demographic data. Categorical variables were presented as percentages or proportions; continuous variables were presented as mean, along with 95% confidence limits. We tested the association between variables with the chi-square test or Fisher’s exact test (for categorical variables) and Student's t-test (for continuous quantitative variables). Subsequently, the odds ratio was calculated for the presence of comorbidities and the use of various antibiotics and *Candida* colonisation. All statistical analyses were performed using Epi Info (version 7.2.5.0; Centers for Disease Control and Prevention, Atlanta, GA).

## Results

A total of 67 patients were enrolled in the study, of whom 37 (55.2%) were male and 30 (44.8%) were female. The age of the patients ranged from 21 to 82 years, with a mean of 50.7 years. Out of the total 67 participants, 39 (58.20%) had one or multiple comorbidities such as hypertension, diabetes, or some known kidney/liver/heart/lung disease. Thirty (44.8%) of 67 patients were started on one or more antibiotics. Sixteen patients were on more than one antibiotic treatment. Piperacillin-tazobactam was the most commonly used antibiotic in the patients (14/30, 46.7%).

Sample collection was done as a composite swab from the patients’ bilateral axilla and groin. Out of the 67 patients enrolled in the study, follow-up samples after one week of admission were only possible for 37 of the patients. Follow-up samples of 30 patients could not be obtained due to discharge (13), death (13), and COVID-19-positive (four) during the first week. In the first sample of patients, 11 (16.4%) out of the total 67 samples showed growth of *Candida* spp. Out of the 37 follow-up samples, only seven (18.9%) showed positive growth. Hence, it was found that 18 (26.9%) out of the 67 patients enrolled in the study showed *Candida* colonisation in one of the samples. None of the participants showed growth in both the first and follow-up samples.

Out of the 18 isolates from culture, only one isolate (5.55%) was identified as *C. auris*. It was isolated from the follow-up sample of one patient. The distribution of other *Candida *species is shown in Table 1.

**Table 1:** Distribution of Candida isolates

Candida species	Number
Candida auris	1 (5.55%)
Candida spp. other than Candida auris	17 (94.45%)
Candida glabrata	13 (72.2%)
Candida parapsilosis	3 (16.7%)
Candida tropicalis	1 (5.55%)
Total	18

The participants whose samples tested positive for *Candida *colonisation were seen for the presence of comorbidity and previous use of antibiotics. Comorbidities and antibiotic usage were more commonly seen in patients with colonisation but were statistically insignificant. On analysis of individual antibiotics usage, meropenem and amoxicillin-clavulanic acid were significantly associated with *Candida *colonisation (Table 2).

**Table 2:** Analysis of comorbidities and use of antibiotics as a risk factor for Candida colonisation

Risk factors	Patients with Candida colonisation (N=18)	Patients without Candida colonisation (N=49)	Odds ratio	P value
Presence of Comorbidities	11 (61.11)	28 (57.14)	1.17	0.77
Use of at least one antibiotics	9 (50.00)	21 (42.86)	1.33	0.6
Use of more than one antibiotics	6 (33.33)	10 (20.41)	1.95	0.27
Individual Antibiotic Analysis				
Piperacillin - Tazobactam	3 (16.67)	11 (22.45)	0.69	0.6
Meropenem	3 (16.67)	1 (2.04)	9.6	0.02
Azithromycin	2 (11.11)	3 (6.12)	1.91	0.49
Ceftriaxone	3 (16.67)	1 (2.04)	9.6	0.02
Amoxicillin-clavulinic acid	1 (5.56)	3 (6.12)	0.9	0.93
Anti-tubercular therapy	1 (5.56)	3 (6.12)	0.9	0.93
Colistin	1 (5.56)	1 (2.04)	2.82	0.45

## Discussion

Infection due to *C. auris *has emerged as an important challenge in the management of patients admitted to ICUs in India due to its potential for infection, multidrug resistance, and associated high mortality [1]. *C. auris* candidaemia usually follows colonization; thus, understanding who, among colonized patients, is at greater risk of developing candidaemia may help improve early diagnosis and prevent invasive infection through interventions involving modifiable predictors [12]. There are no recommended guidelines for screening patients for *C. auris*. This study attempted to estimate the burden of *C. auris* colonisation in ICU patients.

The present study revealed *Candida *colonisation in 26.9% of patients, which is much lower than that reported in other studies in which *Candida *colonisation reached 60% [13-15]. The lower colonisation rate in the current study is attributed to the limited number of sampling sites. The aim of the present study was to detect *C. auris* colonisation, so a composite swab of the patient’s bilateral axilla and groin was taken. In this study, *Candida glabrata *was the most common *Candida *species responsible for colonisation, which is similar to the results of a study performed by Azim et al. [16]. In contrast, a study done by Lau et al. [15] across seven Australian ICUs found that 58% of patients were colonised with *Candida *species, with *Candida albicans* being the most prevalent. In comparison, the Indian subcontinent shows a higher prevalence of non-albicans *Candida *colonisation. In our study, 50% of patients with *Candida *colonisation had a history of antibiotic use, aligning with other studies [17].

The presence of *C. auris* in only one sample is an important finding in this study, indicating a lower detection rate of *C. auris *colonisation at our centre. The patient who showed *C. auris* colonisation in the present study was a male patient in the age group of more than 60 years, having multiple comorbidities, and was exposed to the use of multiple antibiotics, including piperacillin-tazobactum and meropenem. The patient also showed growth of *C. auris* from his blood culture. In a study by Heindel et al. [18] in Germany, no colonisation was detected among patients with a history of previous stay or medical treatment abroad who were screened for *C. auris* upon admission. These results may not apply universally to all healthcare facilities due to variations in *Candida* species prevalence across different geographic regions. It could be beneficial for institutions to develop screening protocols that can be rapidly implemented during outbreaks or for patients with particular risk factors. Identified risk factors include the use of tracheostomies and ventilators, presence of a percutaneous endoscopic gastrostomy tube, prior antibiotic use, ICU admission, and comorbidities such as diabetes mellitus, chronic kidney disease, and lung disease [19,20]. In regions with a high prevalence of *C. auris* or in outbreak situations, the implementation of a rapid and automated molecular surveillance admission screening may prevent the spread of *C. auris* [21]. In regions with low prevalence of *C. auris*, routine admission screening may not be necessary. However, for high-risk patients, identifying *Candida *species from clinical samples at the species level is advisable, especially for non-albicans species, using MALDI-TOF [22].

The limitations of this study include its single-centre nature, small sample size, unavailability of MALDI-TOF MS, and lack of follow-up of patients who were positive for *Candida *colonisation. Furthermore, the availability of molecular assays for *C. auris* screening might have increased the sensitivity of screening.

## Conclusions

Our study indicates a low colonisation rate of *C. auris* among ICU patients in our tertiary care hospital. While overall *Candida* colonisation was observed in approximately one-quarter of patients, *C. auris* was identified in only a single case. These findings suggest that routine screening for *C. auris* may not be warranted in settings with low endemicity. However, given the potential for rapid transmission and high mortality associated with* C. auris* infections, targeted surveillance in high-risk patients and continued vigilance remain essential. Further large-scale, multicentre studies incorporating molecular diagnostic methods are needed to better define colonisation dynamics and guide effective infection control strategies.
